# Supplementary material for: EEG Alpha Band Responses Reveal Amplification Benefits in Infants with Hearing Loss
Source: Children (Basel). 2023 Mar 21;10(3):600. doi: 10.3390/children10030600 (PMC10047398; doi:10.3390/children10030600)
Supplement: Supplementary file 1 [file children-10-00600-s001.zip › children-2242379-supplementary.pdf]

children

MDPI

Supplementary Materials

EEG Alpha Band Responses Reveal Amplification Benefits in Infants with Hearing Loss

Kristin Uhler [1,*,†], Daniel J. Tollin [2] and Phillip M. Gilley [1,3,†]

[1] Department of Physical Medicine and Rehabilitation, University of Colorado Anschutz School of Medicine & Children's Hospital Colorado, Aurora, CO 80045, USA
[2] Department of Physiology and Biophysics, University of Colorado Anschutz School of Medicine, Aurora, CO 80045, USA
[3] Institute of Cognitive Science, University of Colorado Boulder, Boulder, CO 80309, USA
* Correspondence: kristin.uhler@cuanschutz.edu.
† These authors contributed equally to this work.

Hearing Aid Verification

Participant hearing aids were programmed to prevent unnecessary signal distortion and managing audiologists confirmed that all participants' hearing aids were programmed using Desired Sensation Level v5.0.1 Participant's match to Desired Sensation Level targets was used to categorize how well hearing aids were fit. Fit was considered poor if at least one hearing aid deviated from the prescriptive target by ≥ 5dB at 3 frequencies.2,3 The majority of infants' hearing aids met prescriptive gain targets (n = 35), four infants hearing aids did not meet prescriptive gain targets, and two could not be measured due to real ear equipment malfunctioning (Supplementary Table S1). Of the four children who did not meet the prescriptive gain targets, two had excellent audibility per aided speech intelligibility and two likely did not meet targets due to moderately severe to profound degrees of hearing loss.

Hearing aids were assessed using an electroacoustic test box measure before laboratory testing to ensure proper function. Verification of amplification was done using simulated real-ear response measurements which were compared to age-specific Desired Sensation Level targets using real-ear to coupler differences. When real-ear to coupler differences could not be recorded from at least one ear, average values for age specific real-ear to coupler differences were substituted. Aided speech intelligibility index is used to evaluate hearing aid programming based on hearing thresholds by either measuring output of the hearing aid in a child's ear canal or in a test box. The hearing aid output is compared to standard, age-based output targets measured in response to a standardized speech signal. 4 The aided speech intelligibility was automatically calculated at 50-, 60-, and 70-dB SPL using the Audioscan Verifit. Aided speech intelligibility considers the speech spectrum (0 = no access to speech spectrum; 1 = full access to the speech spectrum) that is accessible for participants given their hearing thresholds, ear canal size and shape, and their hearing aid output as measured in the child's ear canal in dB SPL. Because our stimuli were presented at 70 dB SPL, we used the output at that level for aided speech intelligibility, which ranged from 0.45 to 0.95 (M = 0.82, s.d. = 0.11; see Supplementary Table S1).

Table S1. EEG alpha band responses reveal amplification benefit in infants with hearing loss: Hearing aid details.

ID	Sex	Age ID (days)	Age HA Fit (mo)	Age at MMR (mo)	HA Make	HA Model	Better Ear	PTA	Aided SII at 70 dB
36	M	37	1.91	3.84	Phonak	Sky B70P	Left	20	0.93
12	M	48	2.33	2.89	Oticon	SenseiPro90	Left	23	0.86
35	F	105	4.14	4.70	Phonak	SkyB70P	Right	24	0.89
8	M	36	1.84	2.43	Phonak	SkyV70P	Right	26	0.93
15	F	56	3.48	4.60	Phonak	SkyV70P	Left	26	0.89
39	F	18	1.28	1.90	Phonak	Sky V50 P	Left	26	0.89
17	F	19	1.12	1.74	Phonak	SkyV70P	Left	28	0.90
14	F	36	2.43	3.61	Phonak	SkyV70M	Right	29	0.85
27	F	41	3.38	3.51	Phonak	SkyB50P	Right	29	0.86
18	M	60	2.17	3.71	Phonak	SkyV70P	Left	31	0.86
7	M	31	1.78	1.81	Oticon	Sensei Pro	Right	33	ER
9	F	66	3.78	5.09	Phonak	SkyV70P	Left	34	0.95
33	F	47	2.96	3.15	Oticon	SenseiPro90	Right	34	0.89
25	M	43	2.99	5.91	Phonak	SkyV70P	Left	36	0.86
30	M	26	2.46	3.12	Oticon	SenseiPro90	Left	36	0.86
3	M	21	2.23	2.92	Phonak	SkyV50P	Left	38	0.88
4	M	59	6.17	6.31	Phonak	SkyV50M13	Right	39	0.95
20	M	26	2.76	4.24	Oticon	SenseiPro90	Left	40	0.84
26	F	46	2.43	3.94	Phonak	SkyV70M	Left	41	0.85
37	M	39	1.84	3.55	Phonak	Sky B70M	Right	41	0.83
1	F	43	2.10	2.43	Oticon	SenseiPro90	Right	43	0.89
29	F	59	3.35	4.99	Phonak	SkyV70SP	Left	43	0.83
10	M	20	3.12	3.62	Phonak	SkyV70P	Left	44	0.80
11	F	36	2.43	3.98	Phonak	SkyV70P	Left	44	0.82
19	F	23	2.23	2.69	Phonak	SkyV50P	Right	44	0.87
5	M	47	3.16	4.11	Phonak	Nios SIII	Right	46	0.84
34	F	74	4.01	4.70	Phonak	SkyB70M	Right	46	0.82
32	F	35	1.84	2.63	ReSound	UPS777DLW	Left	49	0.82
24	M	47	2.69	5.49	Phonak	SkyV50SP	Right	50	0.77
38	M	52	2.86	2.96	Phonak	Sky B70P	equal	51	0.78
28	F	35	2.73	5.62	Phonak	SkyV70P	Right	53	0.82
13	M	74	2.69	4.01	Phonak	SkyV70P	Left	54	0.78
23	M	44	2.27	5.75	Phonak	Sky V70P	Right	55	0.86
22	F	35	2.04	3.55	Phonak	SkyV70M	Left	56	0.77
31	F	36	4.83	5.52	Phonak	SkyB70P	Left	59	0.81
16	M	24	2.37	3.38	Phonak	SkyV70SP	Right	61	0.71
41	M	33	1.61	4.07	Phonak	Sky B50-UP	Right	75	0.45
2	F	42	1.84	2.56	Phonak	SkyV70	Right	78	ER
6	F	25	3.88	4.63	Phonak	SkyV90SP	Right	85	0.52
21	M	27	2.60	4.30	Phonak	Naida SIII UP	Left	95	0.57
40	F	38	2.66	3.88	Oticon	Opn 1 Play BTE	Left	38	0.81

*Note: *ID* = participant identifier, *Age ID* = age at identification of hearing loss in days, *Age HA Fit* = age at hearing aid (HA) fit in months, *Age at MMR* = age at MMR testing in months, *Better Ear* = better hearing ear based on four frequency pure tone average (*4-Freq PTA*, in dB eHL), estimated sensation level (SL) and aided speech intelligibility (*Aided SII*) measured at 70 dB. For two subjects

there was an equipment malfunction (EM) and the aided speech intelligibility could not be measured at time of MMR. Subjects in blue did not have usable EEG data.

Disclaimer/Publisher's Note: The statements, opinions and data contained in all publications are solely those of the individual author(s) and contributor(s) and not of MDPI and/or the editor(s). MDPI and/or the editor(s) disclaim responsibility for any injury to people or property resulting from any ideas, methods, instructions or products referred to in the content.
